# Lifetime prevalence of cancer in Germany between 2010 and 2019: An analysis based on aggregated data

**DOI:** 10.1371/journal.pone.0334637

**Published:** 2025-11-03

**Authors:** Kira Baginski, Dina Voeltz, Claudia Hornberg, Claudia Kohring, Annika Hoyer

**Affiliations:** 1 Biostatistics and Medical Biometry, Medical School OWL, Bielefeld University, Bielefeld, Germany; 2 Department of Environmental Health Sciences, Medical School OWL, Bielefeld University, Bielefeld, Germany; 3 Department of Epidemiology and Health Care Atlas, Central Research Institute of Ambulatory Health Care in Germany, Berlin, Germany; University Medical Center Hamburg-Eppendorf: Universitatsklinikum Hamburg-Eppendorf, GERMANY

## Abstract

**Background:**

Many cancer survivors (i.e., individuals alive who have been diagnosed with cancer at some point in their lives) remain under surveillance by doctors and medical professionals regardless of the outcome of their treatment as they face the risk of recurrence and/or encounter symptoms and other health-related issues. With advancements in cancer early detection, diagnostics, and treatment strategies, along with an increasingly older population, the number of cancer survivors is steadily increasing. Since information on epidemiological indicators of cancer survivors is limited in Germany, the present study aims to estimate its prevalence between 2010 and 2019.

**Methods:**

The annual age- and sex-specific lifetime prevalence of cancer was estimated using a partial differential equation. The calculation was based on comprehensive data obtained from the Centre for Cancer Registry Data (Zentrum für Krebsregisterdaten, ZfKD), the Central Research Institute of Ambulatory Health Care in Germany (Zentralinstitut für die kassenärztliche Versorgung, Zi), and the German Federal Statistical Office.

**Results:**

Our estimates indicate a rise in lifetime prevalence of cancer between 2010 and 2019 across all ages up to 85, particularly pronounced among older individuals. By 2019, the highest prevalence was estimated to be 49% of males and 28% of females at age 85. In contrast, childhood cancer was relatively rare, affecting less than 0.3% of girls and boys until the age of 18. Generally, the prevalence of cancer was higher among males than females. Overall, the number of cancer survivors aged up to 85 was estimated to increase from 2.51 million in 2010 to over 5.07 million in 2019.

**Conclusions:**

The findings indicate a substantial rise in the lifetime prevalence of cancer in 2019 compared to 2010. Considering a cancer patient as cured based on a specific timeframe may lead to an underestimation of the future demand for healthcare resources.

## Introduction

Over the past 25 years, it is estimated that more than 4.5 million individuals in Germany have been diagnosed with cancer. The total number of living individuals in Germany who have ever received a cancer diagnosis may be approximately 10% higher. Furthermore, Germany reports approximately 500,000 new cancer cases annually [1]. With the demographic aging of the population as well as increases in cancer survival due to advances in early detection and treatment, it is expected that the number of individuals affected by cancer will continue to rise [1,2]. Over the past few decades, cancer has become a notable contributor to mortality in Germany, making it the second leading cause of death [3]. Cancer is not solely linked to a high mortality rate but is also associated with substantial expenses. In 2018, the total cost of cancer in Europe reached €199 billion, with Germany accounting for €47 billion [4].

With the increase in survival probabilities, many cancer survivors (i.e., individuals alive who have received a cancer diagnosis at some point in their lives) experience symptoms and emerging health problems that may arise from cancer itself or its primary treatments like surgery, chemotherapy, and radiation [5–8]. These problems encompass both physical and psychosocial aspects, which may negatively affect quality of life [6,9,10]. This is supported by the fact that cancer survivors may continue to deal with the emotional or physical aftermath and thus may still identify themselves as “cancer patients” [11]. Additionally, post-treatment individuals are at a high risk of cancer recurrence throughout their lifetime, with the level of risk influenced by factors such as type of cancer, treatment methods, and age at diagnosis [8]. Consequently, it is expected that the costs associated with cancer treatment, management, and aftercare will place an increasing burden on the healthcare system. An accurate surveillance of cancer survivors is of high importance including evaluating the overall impact of cancer on healthcare systems. Epidemiological measures, such as the prevalence and incidence, are crucial estimates that can inform preventive measures. At least 30% of cancer occurrences worldwide could be avoided if such preventive measures were implemented [12]. Therefore, ensuring the accuracy of epidemiological measures is crucial since any inaccuracies can hinder risk mitigation efforts, waste resources, and undermine the effectiveness of prevention activities and disease management programs.

Even though several institutions in Germany provide cancer data, there is limited information available regarding the number of cancer survivors corresponding to the lifetime prevalence of cancer. Therefore, the present study aims to estimate the number of individuals aged 0–85 within the German population who have received a cancer diagnosis at some point in their lives.

## Materials and methods

We used mathematical relations between prevalence, incidence, and mortality to assess the age- and sex-specific lifetime prevalence of cancer for individuals aged 0–85 in Germany from 2010 to 2019. For this purpose, we employ a partial differential equation (PDE) that has been previously proposed for estimating the prevalence of chronic diseases [13–18]. Our analysis was grounded in data on cancer prevalence, cancer incidence, the general mortality of the German population, and the mortality rate among people with cancer [2,19,20]. Except cancer prevalence, all data were publicly available.

### Definition of lifetime prevalence

Lifetime prevalence is defined as the proportion of a population that has, at least once throughout their previous lifetime, experienced a particular health event, risk factor, or disease. Here, we are specifically referring to cancer. Hence, in line with Wronski [8], we refer to the affected individuals as cancer survivors, indicating those who are currently alive and have received a cancer diagnosis at some point in their lives. There is no requirement for a specific period of time to have elapsed for someone to be considered a cancer survivor. This definition highlights the journey of individuals who have faced cancer, regardless of the outcome (i.e., still alive and are cancer-free or in ongoing disease and palliative care) of their treatment or the challenges they continue to face. Therefore, in the scope of our research, we define cancer as a chronic condition.

### Statistical analysis

**Fig 1:**
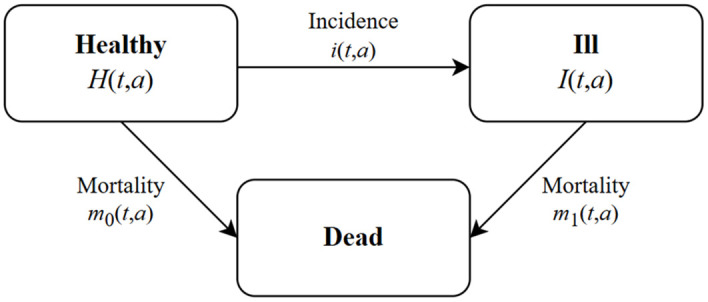
Illness-death model. Every person within a population is either healthy (with respect to the disease of interest), ill or dead. The transitions to another state are represented by the incidence rate *i*, the mortality of the non-diseased ${𝐦}_{\text{0}}$ and diseased ${𝐦}_{\text{1}}$, depending on calendar time *t* and age *a*.

To estimate the annual age-specific prevalence of cancer survivors, we applied a PDE that has already been used in the context of chronic diseases [13–18]. The theoretical background for the PDE is the Illness-death model (IDM) as depicted in Fig 1. The model consists of three states, namely “healthy” (with respect to the disease of interest), “ill” (prevalent cases), and “dead”, and it is assumed that every individual in the population falls into one of these states.

Initially, all individuals are in the healthy state. With passing time, individuals may become diagnosed with a disease of interest, i.e., cancer, and move to the ill state, or they may directly move to the dead state without ever being diagnosed with cancer. Transition rates between the states are the incidence rate i and the mortality of the non-diseased $m_{\text{0}}$ and diseased $m_{\text{1}}$, each depending on calendar time t  and age a. To enhance readability, the time scales are omitted in the subsequent equations. Since $m_{\text{0}}$ is unknown for the majority of diseases, it has been shown possible to incorporate the mortality rate of the general population m, which is given by $m=p\;\times \;m_{\text{1}}+(\text{1}-p)\times m_{\text{0}}\;$ [13,17].

The annual age-specific prevalence can then be calculated as follows [17]:


$$\begin{array}{c} \left(\partial_{t}+\partial_{a}\right)p=(\text{1}-p)\;\times \;\left\{i-p\;\times \frac{\left(m_{\text{1}}-m\right)}{\;(\text{1}-p)}\;\;\;\right\} \end{array}$$

The equation describes the relation between the temporal change in prevalence $\left(\partial_{t}+\partial_{a}\right)p$, the transition rates, and the prevalence p, where ∂ represents the differential operator [16].

The statistical software R, version 4.4.1 [21], was utilized for all analyses. The source code can be accessed publicly on Zenodo [22].

### Data

**Fig 2:**
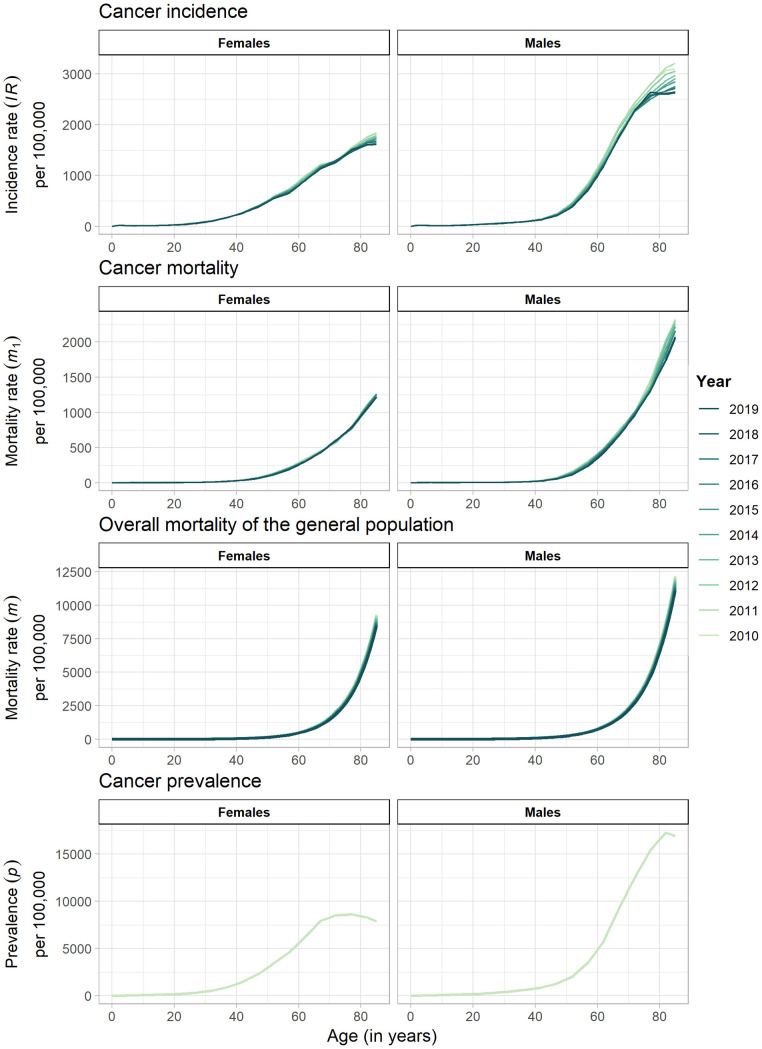
Input data. Data on cancer incidence [19], the cancer-specific mortality rate [19], the overall mortality of the German population [20] between 2010 and 2019 and the prevalence of cancer in 2010 based on [2] in Germany.

More detailed information of already published data on age- and sex-specific prevalence of diagnosed cancer in 2010 (initial values for the PDE) was requested and obtained from the Central Research Institute of Ambulatory Health Care in Germany (Zentralinstitut für die kassenärztliche Versorgung, Zi) [2]. The Zi focused on the administrative annual prevalence of various cancer types and groups among individuals insured under statutory health insurance (SHI) and at least 15 years old during the respective years. Utilizing outpatient claims data from 2010 to 2019, the study of the Zi encompassed around 88% of the total German population, with 87% of this group being 15 years or older [2]. To be defined as prevalent, individuals had to have a confirmed cancer diagnosis (based on the International Classification of Diseases (ICD)-10-GM) documented in at least two quarters of the respective year (M2Q criterion) [2]. The incidence and cancer-specific mortality rates for the same period were taken from the Centre for Cancer Registry Data (Zentrum für Krebsregisterdaten, ZfKD) [19]. The estimation of the nationwide annual incidence of the ZfKD involves adding the cases reported in the cancer registries that met the quality criteria for the diagnosis year to the cases estimated using a mixed Poisson regression model from the remaining registries. Cancer mortality is determined by the annual count of cancer-related deaths as reported in official cause of death statistics [1]. All data were categorized by calendar year, 5-year age groups, and cause of death, coded according to the ICD-10-GM. Only data on cancer incidence, prevalence and mortality for individuals with any cancer diagnosis with ICD-10-GM codes C00 – C95 (excluding non-melanoma skin cancer [C44] and metastases [C77 – C79]) were included. Consistent with previous epidemiological studies on chronic diseases in Germany, we used the age-specific mortality rate of the general population from the 14^th^ population projections provided by the German Federal Statistical Office (FSO) [13–15,20]. To calculate estimates for individual ages from 0 to 85, we linearly interpolated the prevalence, incidence, and mortality data. Fig 2 visualizes all the data utilized. To estimate the number of cancer survivors in Germany during the period from 2010 to 2019, we used the population data from the FSO (see S1 and S2 Figs in the S1 File) [23].

## Results

**Fig 3:**
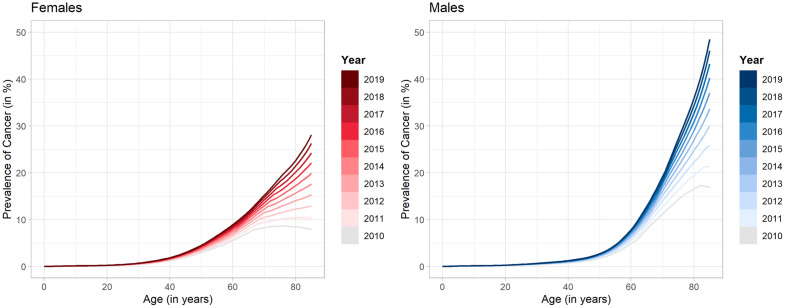
Estimated age- and sex-specific lifetime prevalence of cancer. Estimated lifetime prevalence of cancer in Germany depicted by sex and age for all years from 2010 to 2019. The left plot illustrates the prevalence estimates for females, while the right plot represents the estimates for males.

The estimated sex- and age-specific lifetime prevalence of cancer is shown in Fig 3. Irrespective of sex, the lifetime prevalence follows an exponential growth trend as age increases. The findings indicate an increase in lifetime prevalence from 2010 to 2019 for both sexes over all ages. This trend is particularly noticeable in individuals aged 45 years and above. In general, the prevalence is higher in males than in females. However, between the ages of 30 and 60, females consistently exhibit a higher prevalence than males.

The highest lifetime prevalence in 2010 is estimated among the elderly population, with males aged 82 having the highest estimates at approximately 17%, while females aged 77 in 2010 exhibit the highest prevalence at around 9%. Comparatively, the estimated lifetime prevalence among females and males 50 years of age in the same year is about 3% and 2%, respectively, while that of 20-year-olds is only about 0.2%. Over time, there is a shift in lifetime prevalence towards even older ages. By 2019, the highest lifetime prevalence is seen at age 85 for both males and females, with estimates of around 48% and 28%, respectively. The estimated lifetime prevalence for 50-year-old males in 2019 is approximately 3%, while for females of the same age it is around 5%. On the other hand, the prevalence for 20-year-old males and females is estimated to be approximately 0.3%. When compared to late adulthood cancer, childhood cancer is rare (<0.3% for girls and boys until the age of 18 years). Detailed estimation results of the lifetime prevalence can be found in S1 Table in the S1 File.

**Fig 4:**
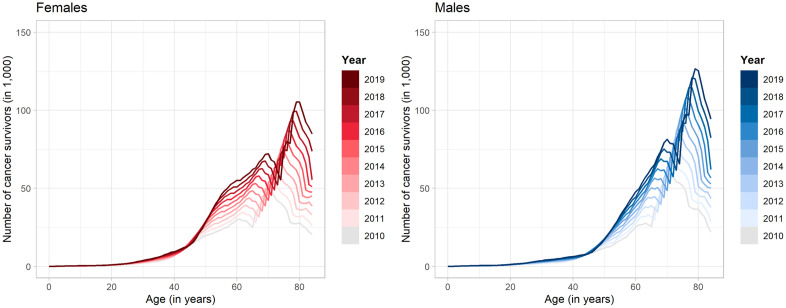
Estimated age- and sex-specific number of cancer survivors. Estimated number of cancer survivors in Germany, categorized by sex and age, for the period from 2010 to 2019. The left plot displays the estimates for females, whereas the right plot shows the estimates for males.

The estimated sex- and age-specific number of cancer survivors based on the lifetime prevalence and the population distribution provided by the FSO is shown in Fig 4. Consistent with lifetime prevalence the results reveal a growing trend in the number of cases from 2010 to 2019 for all ages and both sexes, especially pronounced in the population aged 45 and older.

In 2010, there was a rise in the number of cancer survivors among both males and females up to the age of 60, followed by a subsequent decrease. After the age of 65, there is a rebound in survivor numbers, followed by another decline starting at age 70. As time progresses, it is expected that this trend has shifted towards older populations. Consequently, in 2019, the population of cancer survivors at the age of 70 begins to decrease, followed by an increase from the age of 75, before experiencing another decline after reaching 80. The curve pattern is characteristic and can be linked to the baby boom that occurred following World War II. Overall, the estimate indicates that the number of cancer survivors have increased from 2.51 million in 2010 to over 5.07 million by 2019, with approximately 2.55 million being male and 2.52 million female (see also S3 Fig in the S1 File).

## Discussion

In this study, we aimed to enhance the understanding of the lifetime prevalence of cancer that can be interpreted as prevalence of cancer survivors. To achieve this, we utilized a PDE that had been previously introduced, along with aggregated claims and registry data, to estimate the lifetime prevalence of cancer in Germany for individuals aged 0–85 between 2010 and 2019. This is particularly valuable for public health authorities to be able to optimize intervention programs and allocate resources more efficiently. The findings indicate that the lifetime prevalence of cancer is likely to have more than doubled in 2019 compared to 2010 in almost every age group, particularly among the middle-aged to elderly population. Thus, the number of cancer survivors is estimated to have increased from about 2.51 million in 2010 to approximately 5.07 million by 2019.

The comparison of our estimates and the prevalence of cancer survivors determined in other studies is currently limited to projections for 2014 [24], 2017 [7] and estimates in 2020 [25]. The 2014 projections are derived from the 10-year-prevalence of the ZfKD and data obtained from Sweden [24]. Their estimates indicate that in 2014 around 4.4 million people in Germany were cancer survivors, exceeding our estimate of 3.81 million. Furthermore, their projections suggest that the number of females affected surpasses that of males, with 2.5 million females compared to 1.9 million males [24]. Our findings indicate a different scenario, showing that both males and females were nearly equally impacted, with males totalling 1.93 and females 1.88. Since our estimates only account for individuals up to the age of 85, they likely underestimate the overall population of cancer survivors, especially among females, who generally have a longer life expectancy and lower (all-cause) mortality rate. However, by focusing solely on cancer data from Germany and using administrative annual prevalence rather than interval prevalence, our study increases its validity. Arndt et al. reported that at the end of 2017 there were 4.65 million cancer survivors in Germany, including 2.10 million males and 2.55 million females [7]. Our analysis identified a total of 4.62 million cancer survivors in 2017, comprising 2.33 million males and 2.29 million females. According to the prevalence of cancer survivors in Germany as of Jan 1, 2020 based on the EUROCARE-6 study, which can be compared to our 2019 estimates, there were about 4.8 million cancer survivors. This total comprises 2.5 million females and 2.3 million males [25]. Our analysis for 2019 shows a total of 5.07 million cancer survivors, with around 2.55 million being male and 2.52 million being female. Nevertheless, it is important to note that the German Cancer Registries included in the EUROCARE-6 study only covered 17.2% of the German population, thereby failing to provide a representative sample of the entire population. Overall, our findings are consistent in terms of age with both studies, suggesting that the majority of cancer survivors are aged 75 years or older.

Due to different definitions of prevalence and different methods used to model prevalence, the measures published by Zi and ZfKD as well as our estimates are not directly comparable. The ZfKD employs a prevalence definition that calculates the prevalence within a specific time interval (e.g., 1-, 5-, 10-, 25-year-prevalence), following the method proposed by Pisani et al. [26], where all individuals are excluded outside the time interval. The Zi reports the annual administrative prevalence of all insured individuals who have received a cancer diagnosis, with the additional requirement of the diagnosis being marked as “confirmed” and documented for at least two quarters of the respective year [2]. Consequently, patients who have received a diagnosis that is not yet “confirmed” or is “confirmed” but only documented for a single quarter of the corresponding calendar year were excluded. Additionally, patients with cancer were excluded if their condition did not contribute to healthcare utilization as the treating physician had no reason for documenting the diagnosis. In Germany, claims data are collected primarily for administrative purposes to validate billed medical services, rather than to provide a comprehensive overview of patient history. This may lead to underestimation of cancer patients in outpatient care. Hence, a direct comparison between our point lifetime prevalence, the Zi annual administrative prevalence, and the ZfKD interval prevalence is unfeasible because of the varying prevalence definitions.

In line with Francisci et al. [27], we suggest that the classification of an individual as “cured” from cancer based solely on a specific timeframe should be critically examined. Most survival measures fail to differentiate between truly cured individuals, with a life expectancy comparable to the general population, and not cured individuals who will die of their disease, with the primary goal of treatment being to extend their survival [27]. Nevertheless, the cancers that can be deemed “curable” can be classified as non-chronic. The likelihood of this outcome greatly relies on the timing of detection, as early detection allows for smaller tumors that can often be more easily removed through surgery [28]. However, detecting cancer at such an early stage is extremely challenging, and there remains a risk of recurrence or relapse [8,28]. Additionally, individuals diagnosed with cancer may encounter various physical and psychosocial health issues, stemming from either the cancer itself or the treatment received [6,9,10]. This places an additional burden on the healthcare system while excluding those individuals from prevalence calculations or failing to account for them due to incomplete information would underestimate the healthcare resource needs and impede effective responses.

### Strengths and limitations

A key strength of our study lies in stratifying for both sex and age. Moreover, we were able to use input data from the German context for all measures covering the vast majority of the German population. The main strength of our analysis is the use of a mathematically derived methodology that makes use of relations between prevalence, incidence, and mortality rates which enable us to identify trends in cancer prevalence. The validity of the applied PDE has been mathematically confirmed and further supported by multiple practical validation studies [14–17]. One limitation of the PDE model is the assumption that all individuals initially start in a healthy state. Although this assumption does not hold for every disease, it is reasonable in the context of cancer. Newborns with cancer have not yet been diagnosed and therefore are not captured in cancer registries, resulting in an initial prevalence effectively equal to zero. While this assumption may not be entirely precise, the proportion of individuals born with cancer is very small and can be considered negligible. Another source of inaccuracy in estimation is due to the limitations of the input data, e.g., limited precision and reliability.

Despite its strengths, we acknowledge several limitations primarily associated with the input data. First, the data obtained from the Zi encompasses around 88% of the total German population, specifically those individuals with SHI [2]. Given that our study is based on data from individuals with SHI, future research must investigate whether the same prevalence trend applies to privately insured individuals. The ZfKD has recorded coverage of over 90% throughout the decade [1,29]. While we calculated confidence intervals for our estimates, we did not show them due to their small size, which is attributed to the overall large sample size of the data used. In addition, the ZfKD cancer mortality is based on the number of annual cancer deaths according to the official cause of death statistics. These are linked to inaccuracies that have not yet been determined [30,31]. Second, the categorization used by the Zi and the ZfKD divides data into five-year age groups, except all persons aged 85 and older being grouped in a single age category. This aggregation leads to a high level of uncertainty, as the majority of cancer survivors fall within this age group. The lack of an additional data point beyond the age of 85 prevents interpolation, thereby hindering the ability to accurately assess the age-specific prevalence. Consequently, our analysis was limited to individuals up to the age of 85. Third, due to the utilization of linear interpolation to calculate the prevalence, incidence and cancer mortality for each age, some values may be slightly over- or underestimated, particularly in toddlers, where the prevalence and incidence rates tend to be higher than in older children. However, childhood cancer remains rare, and we therefore assume that this has minimal impact on the overall results. Further, there is a chance that certain individuals might be impacted by multiple types of cancer concurrently or sequentially, potentially skewing our estimates. Another limitation of this study is its reliance on the aforementioned heterogeneous data sources, which stems primarily from constraints in data availability. As a result, the combination of data sources with different population bases introduces a degree of uncertainty in the estimates and may limit the generalizability of the findings. Future research based on harmonized or comprehensive population-based datasets could help to overcome this limitation, enable more robust validation of the findings, and enhance comparability across studies.

Knowing that cancer comprises a range of different types with different symptoms, causes and treatment options, it could be beneficial to examine the different types individually. This approach has the potential to improve our comprehension of the various types of cancer and facilitate more effective treatment.

## Conclusion

We estimated the age- and sex-specific prevalence of cancer survivors aged 0–85 in Germany between 2010–2019. Defining a cancer patient as “cured” solely based on a specific time frame may result in an underestimation of future healthcare resource requirements. Enhancing the availability of data on lifetime prevalence of cancer would be beneficial for comprehending the healthcare demands related to cancer and for implementing targeted preventive measures.

## Supporting information

S1 File Supplementary material. (PDF)
