# Supplementary material for: Lifetime prevalence of cancer in Germany between 2010 and 2019: An analysis based on aggregated data
Source: PLoS One. 2025 Nov 3;20(11):e0334637. doi: 10.1371/journal.pone.0334637 (PMC12582433; doi:10.1371/journal.pone.0334637)
Supplement: S1 File — (PDF) [file pone.0334637.s001.pdf]

Supplementary Material to

Lifetime prevalence of cancer in Germany between 2010 and 2019: An analysis based on aggregated data

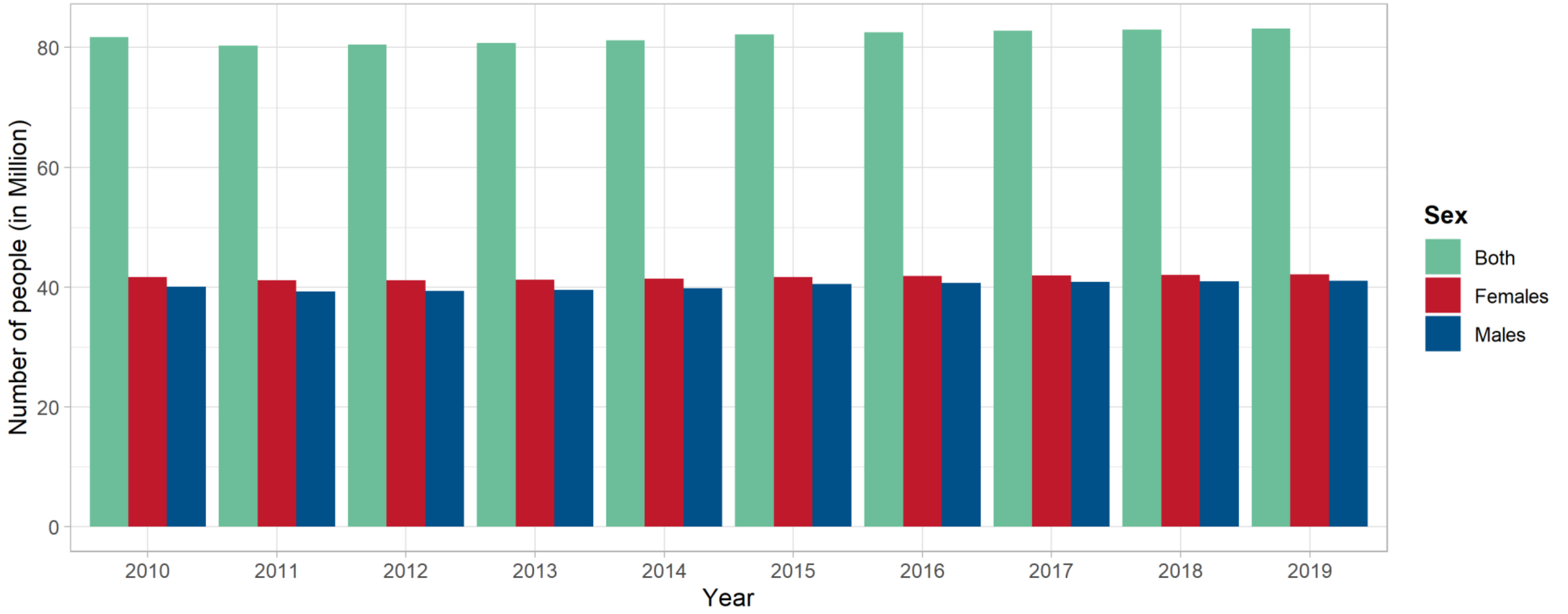

S1 Fig. The population in Germany between 2010 and 2019 for all ages from 0 to 85+ sourced from the database of the Federal Statistical Office (Destatis)[1]

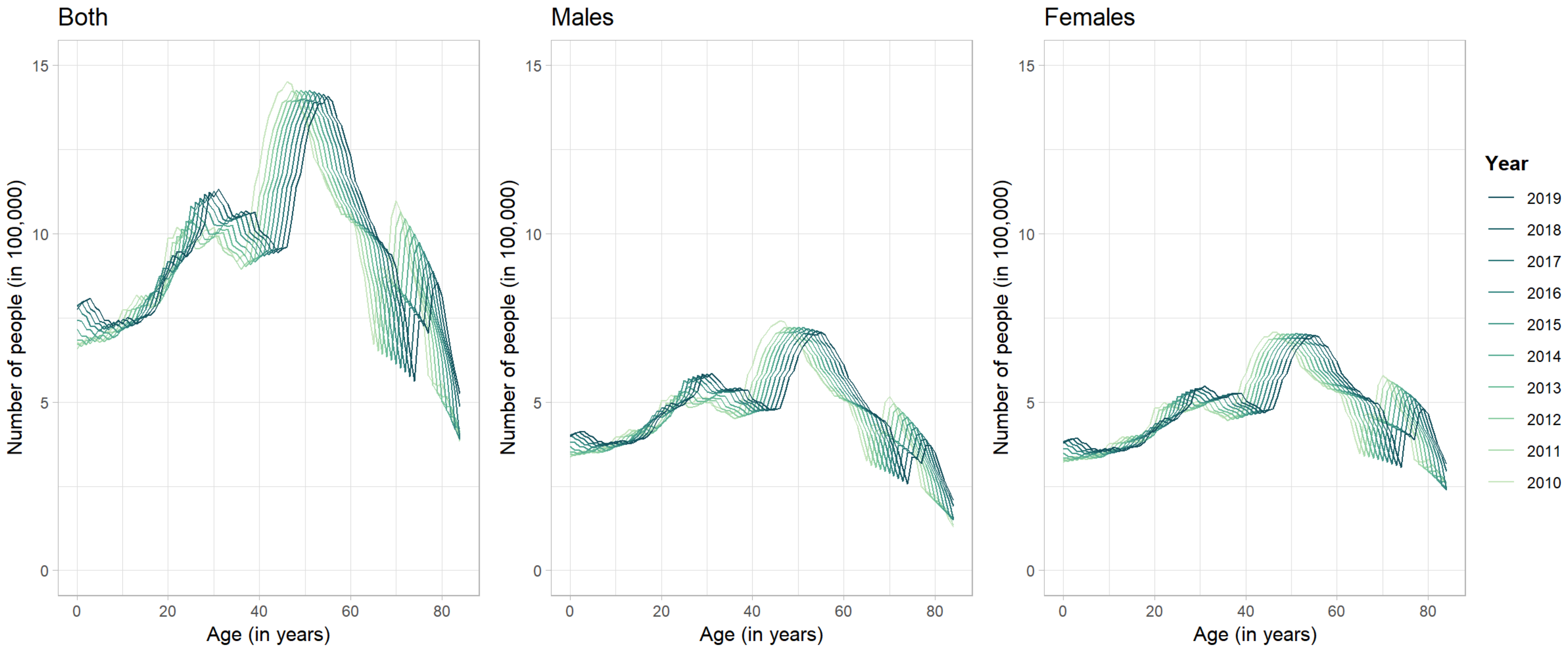

S2 Fig. The age- and sex-specific population in Germany between 2010 and 2019 sourced from the database of the Federal Statistical Office (Destatis)[1]

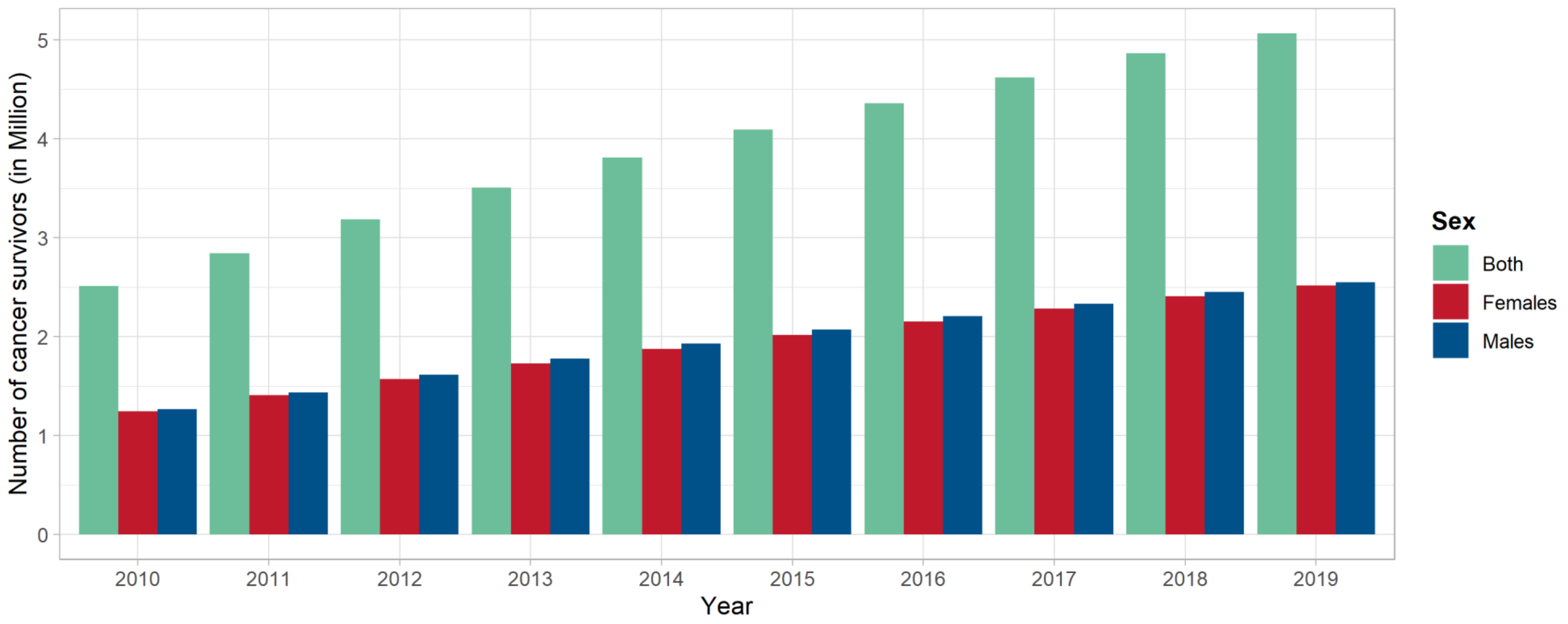

S3 Fig. Estimated case numbers of cancer survivors in Germany between 2010 and 2019 for the age range 0 to 85

Detailed estimation results

S1 Table. Prevalence estimation results Estimated age- and sex-specific prevalence (in %) between 2010 and 2019

		Age																	
**Sex**	**Year**	**0**	**5**	**10**	**15**	**20**	**25**	**30**	**35**	**40**	**45**	**50**	**55**	**60**	**65**	**70**	**75**	**80**	**85**
**Females**	2010	0	0.041	0.083	0.124	0.174	0.275	0.451	0.753	1.248	1.980	2.989	4.148	5.556	7.232	8.286	8.585	8.435	7.893
	2011	0	0.047	0.084	0.132	0.193	0.301	0.497	0.820	1.352	2.145	3.244	4.552	6.070	7.937	9.402	10.040	10.368	10.377
	2012	0	0.056	0.085	0.137	0.208	0.322	0.535	0.873	1.437	2.288	3.464	4.919	6.526	8.578	10.478	11.423	12.242	12.860
	2013	0	0.066	0.086	0.140	0.220	0.338	0.565	0.913	1.501	2.403	3.648	5.249	6.920	9.154	11.520	12.726	14.041	15.315
	2014	0	0.072	0.088	0.143	0.231	0.362	0.603	0.977	1.591	2.541	3.860	5.552	7.341	9.707	12.284	13.887	15.680	17.636
	2015	0	0.069	0.092	0.144	0.239	0.382	0.635	1.031	1.668	2.659	4.043	5.819	7.721	10.199	12.988	15.022	17.250	19.900
	2016	0	0.069	0.098	0.145	0.246	0.397	0.661	1.076	1.733	2.753	4.194	6.049	8.064	10.629	13.622	16.116	18.737	22.095
	2017	0	0.069	0.107	0.146	0.249	0.407	0.680	1.113	1.783	2.827	4.312	6.237	8.371	11.005	14.199	17.171	20.147	24.211
	2018	0	0.069	0.119	0.147	0.249	0.412	0.690	1.144	1.817	2.886	4.407	6.387	8.649	11.335	14.724	18.197	21.474	26.254
	2019	0	0.068	0.125	0.149	0.248	0.416	0.706	1.181	1.876	2.968	4.529	6.567	8.907	11.703	15.217	18.943	22.642	28.097
**Males**	2010	0	0.044	0.088	0.132	0.192	0.284	0.410	0.566	0.780	1.128	1.754	2.945	4.876	7.901	11.235	14.250	16.533	16.913
	2011	0	0.052	0.092	0.142	0.209	0.310	0.446	0.619	0.852	1.239	1.959	3.299	5.495	8.775	12.698	16.286	19.460	21.359
	2012	0	0.062	0.095	0.148	0.225	0.332	0.479	0.664	0.916	1.328	2.111	3.567	6.002	9.514	14.034	18.178	22.234	25.778
	2013	0	0.074	0.099	0.153	0.237	0.353	0.510	0.703	0.969	1.393	2.215	3.744	6.378	10.082	15.210	19.901	24.813	30.076
	2014	0	0.082	0.104	0.156	0.252	0.379	0.544	0.744	1.027	1.479	2.355	3.996	6.798	10.754	16.149	21.409	26.993	33.648
	2015	0	0.080	0.110	0.160	0.262	0.400	0.575	0.784	1.080	1.555	2.466	4.196	7.136	11.315	16.942	22.796	29.032	37.034
	2016	0	0.080	0.117	0.164	0.270	0.417	0.600	0.821	1.127	1.617	2.549	4.338	7.394	11.783	17.602	24.071	30.928	40.219
	2017	0	0.080	0.127	0.168	0.275	0.428	0.618	0.853	1.167	1.667	2.609	4.430	7.570	12.164	18.151	25.244	32.700	43.228
	2018	0	0.080	0.140	0.172	0.278	0.436	0.634	0.880	1.203	1.710	2.650	4.482	7.672	12.453	18.600	26.334	34.379	46.007
	2019	0	0.078	0.148	0.175	0.279	0.447	0.654	0.910	1.241	1.760	2.708	4.569	7.850	12.811	19.205	27.247	35.898	48.482

References

1. Federal Statistical Office of Germany. Current updating of population figures: GENESIS-Online database. https://www.destatis.de/EN/Themes/Society-Environment/Population/Current-Population/_node.html. Accessed 09-19-2024.
